# Addiction systems antagonize bacterial adaptive immunity

**DOI:** 10.1093/femsle/fnz047

**Published:** 2019-03-05

**Authors:** Lisa van Sluijs, Stineke van Houte, John van der Oost, Stan JJ Brouns, Angus Buckling, Edze R Westra

**Affiliations:** 1 Laboratory of Microbiology, Wageningen University, Stippeneng 4, 6708 WE, Wageningen, the Netherlands; 2 Environment and Sustainability Institute, University of Exeter, Penryn campus, Penryn, TR10 9FE, UK

**Keywords:** CRISPR, TA, toxin, adaptive immunity, plasmid, bacteria

## Abstract

CRISPR-Cas systems provide adaptive immunity against mobile genetic elements, but employment of this resistance mechanism is often reported with a fitness cost for the host. Whether or not CRISPR-Cas systems are important barriers for the horizontal spread of conjugative plasmids, which play a crucial role in the spread of antibiotic resistance, will depend on the fitness costs of employing CRISPR-based defences and the benefits of resisting conjugative plasmids. To estimate these costs and benefits we measured bacterial fitness associated with plasmid immunity using *Escherichia coli* and the conjugative plasmid pOX38-Cm. We find that CRISPR-mediated immunity fails to confer a fitness benefit in the absence of antibiotics, despite the large fitness cost associated with carrying the plasmid in this context. Similar to many other conjugative plasmids, pOX38-Cm carries a CcdAB toxin–anti-toxin (TA) addiction system. These addiction systems encode long-lived toxins and short-lived anti-toxins, resulting in toxic effects following the loss of the TA genes from the bacterial host. Our data suggest that the lack of a fitness benefit associated with CRISPR-mediated defence is due to expression of the TA system before plasmid detection and degradation. As most antibiotic resistance plasmids encode TA systems this could have important consequences for the role of CRISPR-Cas systems in limiting the spread of antibiotic resistance.

## Teaser

A cost of CRISPR immunity due to addiction systems.

## INTRODUCTION

Prokaryotes often carry multiple immune systems (Labrie, Samson and Moineau 2010; Doron *et al*. 2018), including a highly sophisticated adaptive immune system known as CRISPR-Cas (Clustered Regularly Interspaced Short Palindromic Repeats—CRISPR-associated), reviewed in (Marraffini 2015). This system functions by integrating sequences of viruses, plasmids and transposable elements (Barrangou *et al*. 2007; Bikard *et al*. 2012; Lopez-Sanchez *et al*. 2012) (known as spacers) into CRISPR loci, which subsequently provide immunity against re-infection (Barrangou*, et al*. 2007; Brouns *et al*., 2008; Tyson and Banfield, 2008; Datsenko *et al*. 2012; Swarts *et al*. 2012; Yosef, Goren and Qimron 2012; van Houte, Buckling and Westra 2016a). Conjugative plasmids often carry antibiotic resistance genes and therefore play a crucial role in the spread of antibiotic resistance (Maiden 1998; Dionisio *et al*. 2002; Svara and Rankin, 2011; Carattoli, 2013). Whether CRISPR-dependent immunity to plasmids is important in limiting the spread of antibiotic resistance (Marraffini and Sontheimer, 2008 and Palmer and Gilmore, 2010; Gophna *et al*. 2015) depends on the efficacy of the CRISPR-Cas immune system (Hullahalli *et al*. 2018), and on the fitness cost associated with carrying the plasmid (in the absence of antibiotics) and the cost of resistance associated with CRISPR-immunity. Experimental observations (Jiang *et al*. 2013; Vercoe *et al*. 2013) and theory predicts that CRISPR-Cas systems can degenerate if they carry a cost (Levin 2010; Weinberger, Wolf and Lobkovsky 2012; Iranzo *et al*. 2013). While large costs are likely when the CRISPR-Cas system behaves maladaptively, such as autoimmunity (Stern *et al*. 2010; Vercoe *et al*. 2013) and the prevention of beneficial infection (Bikard *et al*. 2012; Jiang *et al*. 2013), there may also be costs when the system prevents infection by costly genetic elements, for example due to immunopathological effects or energetic costs of immune activation (Vale *et al*. 2015; Westra *et al*. 2015; Westra *et al*. 2016; van Houte *et al*. 2016b). Here, we investigate this possibility using *Escherichia coli* and the conjugative F-plasmid pOX38-Cm. Our data show that CRISPR-mediated immunity against this costly plasmid is associated with a fitness cost under non-selective conditions. Our data further suggest that this cost of immunity may not only result from energetic costs, but is caused by a plasmid-encoded CcdAB toxin–antitoxin (TA) addiction system (Ogura and Hiraga 1983; Jaffe, Ogura and Hiraga 1985; Bahl, Hansen and Sorensen 2009), which plays a critical role in avoiding plasmid curing (Hayes 2003). Hence, TA systems may limit the evolution of bacterial adaptive immunity against plasmids, which could have important consequences for the spread of antibiotic resistance.

## METHODS

### Bacterial strains

*Escherichia coli* K12 Δ*hns* (BW25113) strains, which have a constitutively active CRISPR-Cas system (Pul *et al*. 2010; Westra *et al*. 2010) were used as recipient cells. These strains, which were obtained from the KEIO collection, were cured from the kanamycin resistance cassette using FLP recombinase (Datsenko and Wanner 2000) and were engineered to carry synthetic CRISPR loci, sequences of which can be found in Table S2 (Supporting Information). *Escherichiacoli* MC4100 carrying pOX38-Cm was used as the donor strain.

### Cloning of spacers, lacZ and CcdA

Spacers, *lacZ* and *ccdA* were cloned into the recombination cassette located on the previously described plasmid pRECOMB-Cr2.1(Westra *et al*. 2010). Spacer-containing DNA fragments from plasmid pWUR693 and pWUR700 (Westra *et al*. 2013) were cloned using the BamHI and EcoRI restriction sites of pRECOMB-Cr2.1. The *lacZ* and *ccdA* genes were PCR amplified from the *E. coli* K12 W3110 genome and plasmid pOX38-Cm, respectively (Figure S1, Tables S1 and S2, Supplementary Information) and cloned using restriction enzymes NotI and KpnI. Resulting plasmids were used as a template for PCR amplification using primers BG4452 and BG4453 (Figure S1, Tables S1 and S2, Supplementary Information) and the amplicon was subsequently gel purified. *Escherichia coli* Δ*hns* cells containing the plasmid pKD46 were transformed with the amplicon (Datsenko and Wanner 2000), after which the bacteria were plated on LB agar containing kanamycin (50 mg/L) to select for recombinants. Plated bacteria were grown at 37°C overnight to cure the cells from pKD46. Recombination was confirmed using colony PCR and Sanger sequencing (GATC Biotech, Germany).

### Growth measurements

Growth curves of *E. coli* K12 Δ*hns* (BW25113) and *E. coli* K12 Δ*hns* (BW25113) carrying pOX38-Cm were measured as follows. Bacteria were inoculated 1:100 in 1 L fresh LB medium from overnight cultures containing the same optical density and grown at 37°C while shaking at 180 rpm (four replicas per treatment). The optical density (OD_600_) of the cultures was measured every 30 min. At each of these time points a sample of 10 or 20 mL was taken. Cells were washed with Millipore water and dried overnight at 130°C. The dry weight of the bacteria was measured of every sample to determine the specific growth rate (in gram new cells·gram cell^−1^·hr^−1^). The specific growth rate was determined for the log phase of the growth curve and used as a measurement of bacterial fitness.

### Competition experiments

Competition experiments were inoculated from overnight cultures grown at 37°C with equal optical densities. Competition experiments were performed in microcosms (6 mL LB medium in 30 mL glass vials, 6 replicas per treatment), containing 60 μL of every culture used in the experiment. Competition experiments were incubated at 37°C at 100 rpm and a daily transfer of 120 μL of the competition experiments into fresh microcosms was carried out. After 2 days cells were plated on LB agar containing kanamycin (50 mg/L) and X-gal (50 mg/L). Colony counting of blue and white colonies was used to determine relative fitness of CRISPR-immune (white colonies) and CRISPR-sensitive strains (blue colonies). Experiments were performed in presence or absence of pOX38-Cm to measure the effect of plasmid presence on the fitness of the bacterial strains. Statistical analysis was performed using JMP10 Software.

## RESULTS

**Figure 1.**
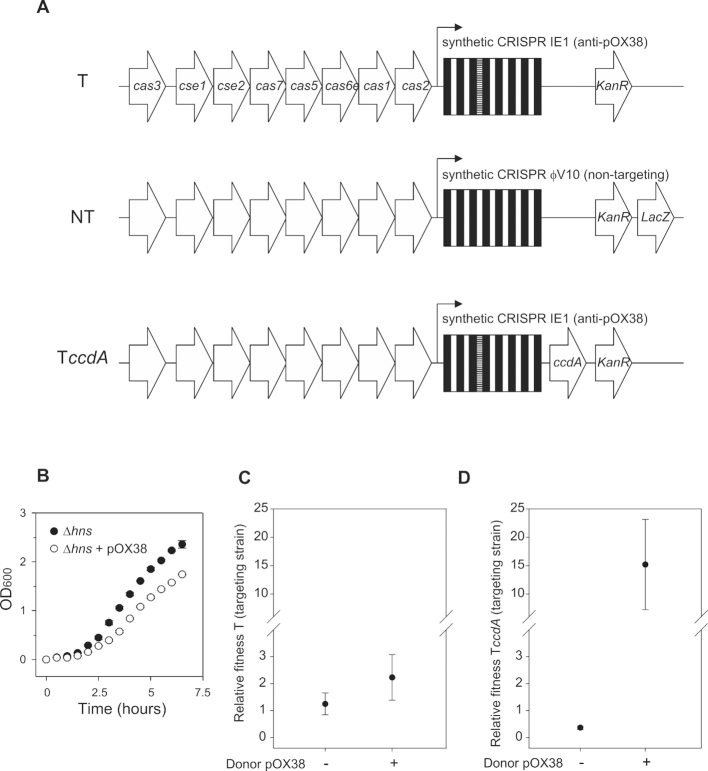
**(A)**, Overview of the engineered CRISPR locus of the T strain (targeting pOX38-Cm), the NT strain (not targeting pOX38-Cm, encoding LacZ) and the T*ccdA* strain (targeting pOX38-Cm, encoding CcdA). Genes are indicated by arrows. The CRISPR locus consists of repeats (black) and spacers (white). The spacer targeting pOX38-Cm is indicated by horizontal stripes. **(B)**, Optical densities at 600 nm (OD_600_) of plasmid-free (Δ*hns*) cells and plasmid-containing (Δ*hns + *pOX38-Cm) cells at different time points after inoculation. Measurements of dry weight were used to determine specific growth rates (g new cells·g cells^−1^·hr^−1^, Table 1). **(C)**, Relative fitness (mean ± 95% CI) of T strain in the absence or presence of the pOX38-Cm donor strain after 2 days of competition with NT strain. **(D)**, Relative fitness (mean ± 95% CI) of T*ccdA* strain in the absence or presence of the pOX38-Cm donor strain after 2 days of competition with NT strain.

It has previously been shown that the Type I-E CRISPR-Cas adaptive immune system of *E. coli* (Fig. 1A) can effectively protect against conjugative transfer of plasmid pOX38-Cm (Westra *et al*. 2013), which is a derivative of the well-studied plasmid F and encodes chloramphenicol (Cm) resistance. Measurements of the specific growth rates of *E. coli* Δ*hns* reveal that carrying plasmid pOX38-Cm reduces growth rates with 27% when antibiotics are absent (Fig. 1B and Table 1; F_1,7_ = 50.67, *P* = 0.0004). Based on the difference in growth rate between plasmid-containing and plasmid-free cells in monoculture, CRISPR-mediated immunity against the plasmid would be expected to result in a large fitness benefit. To measure the relative fitness associated with CRISPR immunity, competition experiments were performed between CRISPR-immune and susceptible *E. coli* K12 derived strains. To this end, the genome of *E. coli* K12 Δ*hns* was engineered to replace the CRISPR 2.1 locus flanking the *cas* genes with synthetic CRISPR arrays that either target (strain T) plasmid pOX38-Cm or that do not target (non-targeting; strain NT) the plasmid (Fig. 1A). After competing strain T and strain NT for two days the resulting relative fitness is approximately one, indicating that the two strains have comparable fitness (Fig. 1C; 1-sample t-test, T_5 _= 1.19, *P* = 0.29). Surprisingly, the presence of a donor strain that carries conjugative plasmid pOX38-Cm did not cause a significant fitness increase of the T strain compared to when the plasmid was absent (Fig. 1C; F_1,11_ = 4.23, *P* = 0.067). These data therefore suggest that the cost of immunity is of the same order of magnitude as the cost of carrying the plasmid.

**Table 1.** Specific growth rates of bacteria with and without plasmid pOX38-Cm.

Strain	Specific growth rate (g new cells*g cells^−1^*hr^−1^)
Δ*hns*	0.90 ± 0.06
Δ*hns* + pOX38-Cm	0.66 ± 0.04

Given the T and NT strains did not differ in fitness in the absence of the plasmid, we hypothesized that the cost of immunity associated with CRISPR-Cas could be due to gene expression from the invading plasmid prior to detection by the immune system, analogous to the expression of anti-CRISPR genes from phage genomes prior to CRISPR-mediated cleavage of the phage genomes (Bondy-Denomy *et al*. 2013; Borges *et al*. 2018; Landsberger *et al*. 2018). Although any of the plasmid genes could contribute to this cost, it is well documented that expression of plasmid-encoded addiction systems would be particularly harmful. Addiction systems prevent plasmid curing, since removal of the plasmid results in rapid depletion of the anti-toxin whereas the toxin will persist for longer periods of time to eventually cause cell death (Gerdes and Maisonneuve 2012; Cook *et al*. 2013). The toxin–anti-toxin (TA) system of plasmid pOX38-Cm is encoded by the *ccdAB* genes; the CcdB toxin is neutralized by the CcdA anti-toxin. In the absence of the short-lived CcdA anti-toxin the CcdB toxin inhibits DNA gyrase, which eventually leads to cell death (Cook *et al*. 2013).

To test the hypothesis that this TA system contributes to the cost of resistance we engineered an *E. coli* strain to express the CcdA anti-toxin from the genome in addition to carrying the T CRISPR (T*ccdA* strain; Fig. 1A). This strain is immune to plasmid pOX38-Cm and to the detrimental effect of toxin CcdB since the toxin is neutralized by CcdA. Competition between the T*ccdA* and the NT strain in the absence of the conjugative plasmid reveals that encoding CcdA on the genome is associated with a fitness cost (relative fitness = 0.37) (Fig. 1D; T_5_ = −19.2, *P* < 0.0001) after two days of competition. By contrast, when competing T*ccdA* and NT for two days in the presence of a donor strain that carries the pOX38-Cm plasmid, the T*ccdA* strain has a large fitness benefit (relative fitness = 15.2) (Fig. 1D; T_5_ = 3.5, *P* = 0.017). Hence, these data demonstrate that expression of the anti-toxin from the bacterial chromosome alleviates the cost of CRISPR immunity, suggesting that TA expression from the plasmid prior to its degradation by CRISPR-Cas immune systems may be an important contributor to the observed cost of immunity.

## DISCUSSION

Costs of resistance potentially have profound effects on co-evolutionary dynamics (Agrawal and Lively 2003; Lopez-Pascua and Buckling 2008;Gomez and Buckling 2011; Buckling and Brockhurst 2012) and are directly responsible for the existence of trade-offs between immunity and other life-history traits (Boots and Begon 1993; Boots and Bowers 2004; Little and Killick 2007; Kempel *et al*. 2011). Our data suggest that TA systems encoded by plasmids may cause CRISPR immunity against an invading plasmid to be costly, due to the time-lag between infection and clearance of the infection during which the TA system may already be expressed. As a result of this cost the CRISPR system may have little net benefit against costly plasmids if they encode TA-systems. This could explain the limited spread and degeneration of CRISPR-Cas systems, and may also help to explain observations of high degrees of susceptibility to costly plasmids in *E. coli* (Touchon *et al*. 2012). Furthermore, many antibiotic resistance genes are carried on conjugative plasmids containing TA systems (Maiden 1998, Dionisio *et al*. 2002; Svara and Rankin 2011; Carattoli 2013). As such, there will be relatively weak selection to resist these plasmids via CRISPR-Cas, even in the absence of antibiotic selection. Our findings also indicate that using CRISPR-Cas as a tool to resensitize bacteria to antibiotics by selectively removing antibiotic resistance-carrying conjugative plasmids (Pursey *et al*. 2018) may be challenging when these plasmids encode TA systems.

## Supplementary Material

Supplemental File
