# Supplementary material for: Addiction systems antagonize bacterial adaptive immunity
Source: FEMS Microbiol Lett. 2019 Mar 5;366(5):fnz047. doi: 10.1093/femsle/fnz047 (PMC6478593; doi:10.1093/femsle/fnz047)
Supplement: Supplemental File [file fnz047_supplemental_file.docx]

**Supplementary information**

**Figure S1.** Primer annealing positions on the recombination cassette pRECOMB-Cr2.1. Primers are described in Table S1.

3

4, 5

1

10

2

**Table S1.** Primers used in this study. Primer annealing positions are shown in Fig. S1.

| *Primer nr.* | *BG number* | *Sequence* | *Position* |
| --- | --- | --- | --- |
| 1 | BG4452 | CGTGCTTGCCGCTGGAGAAATACAA | CRISPR recombination cassette FW |
| 2 | BG4453 | CGGTGTAAAAACCCCTGAGGCAGTA | CRISPR recombination cassette RV |
| 3 | BG4473 | CGAGGCAGATTAGCCATCTGGTTGTTGGAGGTACG | CRISPR recombination cassette FW (start 200 bp downstream primer 1) |
| 4 | BG4457 | TGACGGCGAGAATATGCCGCGAAAG | Spacer IE1 RV |
| 5 | BG4549 | AGAGCAACTGGTATTTCAAATGGCCTGCCGTG | Spacer φV10 RV |
| 6 | BG4514 | CCCGGGGCGGCCGCTTATTTTTGACACCAGACCAACTGGTAATG | NotI site *lacZ* FW |
| 7 | BG4515 | CCCGGGGCGGCCGCGCGCAACGCAATTAATGTGAGTTAGCTCAC | NotI site *lacZ* RV |
| 8 | BG4516 | CCCGGGGGTACCCCATACGAAACGGGAATGCGGTAATTACGCTTTGT | KpnI site *ccdA* FW |
| 9 | BG4517 | CCCGGGGGTACCTCACCAGTCCCTGTTCTCGTCAGCAAAAGAGCCGT | KpnI site *ccdA* FW |
| 10 | BG4455 | CTCAAGCGGATGAGTGACACCGA | *E. coli* genome downstream CRISPR |

**Table S2.** Sequences of synthetic CRISPR arrays.

**Synthetic CRISPR cassette of Cr2.1::φV10**

CTGCAGCGTGCTTGCCGCTGGAGAAATACAACCGCCGGCCCCACCTGAAGATGCACAGCCTGTTGCCATTCCGCTTCCTGTTTCACTGGGAGATGCAGGCCATCGGAGTAGCTGAAATGAGTATGTTGGTCGTGGTCACTGAAAATGTACCTCCGCGCTTACGAGGCAGATTAGCCATCTGGTTGTTGGAGGTACGTGCAGGGGTATATGTAGGTGATGTATCCGCAAAAATTCGTGAAATGATCTGGGAACAAATAGCTGGACTGGCGGAAGAAGGCAATGTAGTGATGGCATGGGCAACGAATACGGAAACGGGATTTGAGTTCCAGACATTTGGGTTAAACAGGCGTACCCCGGTAGATTTGGATGGTTTAAGGTTGGTGTCTTTTTTACCTGTTTGACCATGGAAACAAAGAATTAGCTGATCTTTAATAATAAGGAAATGTTACATTAAGGTTGGTGGGTTGTTTTTATGGGAAAAAATGCTTTAAGAACAAATGTATACTTTTAGAGAGTTCCCCGCGCCAGCGGGGATAAACCGTGGGATGCCTACCGCAAGCAGCTTGGCCTGAAGAGTTCCCCGCGCCAGCGGGGATAAACCGGACCGACGTCCACCGAATTCAGAAAGGACCACGAGTTCCCCGCGCCAGCGGGGATAAACCGCACGGCAGGCCATTTGAAATACCAGTTGCTCTGAGTTCCCCGCGCCAGCGGGGATAAACCGGTTGGCCTTAGTTGGGATCCCTAGGTGCCTGGGAGTTCCCCGCGCCAGCGGGGATAAACCGGCCCATTCCAAAGGGTCAGGTTCGGTGGTTGTGAGTTCCCCGCGCCAGCGGGGATAAACCGGACTTACATTTATAATGCATAATAGGAATCACGAGTTCCCCGCGCCAGCGGGGATAAACCGACGGCTTCATGTTCCTTGTGATGGGCTTTAGGGAGTTCCCCGCGCCAGCGGGGATAAACCGCAGCTCCCATTTTCAAACCCAGGTACCGAAGTTCCTATACTTTCTAGAGAATAGGAACTTCGCATGCCTATTTGTTTATTTTTCTAAATACATTCAAATATGTATCCGCTCATGAGACAATAACCCTGATAAATGCTTCAATAATATTGAAAAAGGAAGAGTATGAGCCATATTCAACGGGAAACGTCTTGCTCTAGGCCGCGATTAAATTCCAACATGGATGCTGATTTATATGGGTATAAATGGGCTCGCGATAATGTCGGGCAATCAGGTGCGACAATCTATCGATTGTATGGGAAGCCCGATGCGCCAGAGTTGTTTCTGAAACATGGCAAAGGTAGCGTTGCCAATGATGTTACAGATGAGATGGTCAGACTAAACTGGCTGACGGAATTTATGCCTCTTCCGACCATCAAGCATTTTATCCGTACTCCTGATGACGCATGGTTACTCACCACTGCGATCCCCGGGAAAACAGCATTCCAGGTATTAGAAGAATATCCTGATTCAGGTGAAAATATTGTTGATGCGCTGGCAGTGTTCCTGCGCCGGTTGCATTCGATTCCTGTTTGTAATTGTCCTTTTAACAGCGACCGCGTATTTCGTCTCGCTCAGGCGCAATCACGAATGAATAACGGTTTGGTTGATGCGAGTGATTTTGATGACGAGCGTAATGGCTGGCCTGTTGAACAAGTCTGGAAAGAAATGCACAAACTTTTGCCATTCTCACCGGATTCAGTCGTCACTCATGGTGATTTCTCACTTGATAACCTTATTTTTGACGAGGGGAAATTAATAGGTTGTATTGATGTTGGACGAGTCGGAATCGCAGACCGATACCAGGATCTTGCCATCCTATGGAACTGCCTCGGTGAGTTTTCTCCTTCATTACAGAAACGGCTTTTTCAAAAATATGGTATTGATAATCCTGATATGAATAAATTGCAGTTTCATTTGATGCTCGATGAGTTTTTCTAACTCGAGGAAGTTCCTATACTTTCTAGAGAATAGGAACTTCGCGGCCGCTCAAGACGCCTTCGCCAACTCCTTCACCAGAGGTAGCATTATCCGCATAACGTCACGGCAGCGACGTTCTATTCTTCCAGGAAGAGCCTTATCAATATGTTGGTGATTATCCAGTCTTACGTCATGCCAGCTATTTCCCGCCGGGAAGGCAGGTGTTTTTGCGCGTTGCTGATAACCATCCTTATTCCCAAGATTCCAGTTAGTCGCTTCCACCGAAAGTACAGCAATGCCCGCTTTGTCGAATATTTCTGCGTCATTACAACACCCAGTGCCTTTCGGATAATTTTTATTCAAACCCGGATTGGTCGTTGCGGCTATTCCGTGACTGCGCGCAATTGCCAGCGCCCTGTCGCGCGTTAATTTCCTTACTGCCTCAGGGGTTTTTACACCGCTGTTGAAAAAGCTT

**Synthetic CRISPR cassette of Cr2.1::φV10 *lacZ***

CTGCAGCGTGCTTGCCGCTGGAGAAATACAACCGCCGGCCCCACCTGAAGATGCACAGCCTGTTGCCATTCCGCTTCCTGTTTCACTGGGAGATGCAGGCCATCGGAGTAGCTGAAATGAGTATGTTGGTCGTGGTCACTGAAAATGTACCTCCGCGCTTACGAGGCAGATTAGCCATCTGGTTGTTGGAGGTACGTGCAGGGGTATATGTAGGTGATGTATCCGCAAAAATTCGTGAAATGATCTGGGAACAAATAGCTGGACTGGCGGAAGAAGGCAATGTAGTGATGGCATGGGCAACGAATACGGAAACGGGATTTGAGTTCCAGACATTTGGGTTAAACAGGCGTACCCCGGTAGATTTGGATGGTTTAAGGTTGGTGTCTTTTTTACCTGTTTGACCATGGAAACAAAGAATTAGCTGATCTTTAATAATAAGGAAATGTTACATTAAGGTTGGTGGGTTGTTTTTATGGGAAAAAATGCTTTAAGAACAAATGTATACTTTTAGAGAGTTCCCCGCGCCAGCGGGGATAAACCGTGGGATGCCTACCGCAAGCAGCTTGGCCTGAAGAGTTCCCCGCGCCAGCGGGGATAAACCGGACCGACGTCCACCGAATTCAGAAAGGACCACGAGTTCCCCGCGCCAGCGGGGATAAACCGCACGGCAGGCCATTTGAAATACCAGTTGCTCTGAGTTCCCCGCGCCAGCGGGGATAAACCGGTTGGCCTTAGTTGGGATCCCTAGGTGCCTGGGAGTTCCCCGCGCCAGCGGGGATAAACCGGCCCATTCCAAAGGGTCAGGTTCGGTGGTTGTGAGTTCCCCGCGCCAGCGGGGATAAACCGGACTTACATTTATAATGCATAATAGGAATCACGAGTTCCCCGCGCCAGCGGGGATAAACCGACGGCTTCATGTTCCTTGTGATGGGCTTTAGGGAGTTCCCCGCGCCAGCGGGGATAAACCGCAGCTCCCATTTTCAAACCCAGGTACCGAAGTTCCTATACTTTCTAGAGAATAGGAACTTCGCATGCCTATTTGTTTATTTTTCTAAATACATTCAAATATGTATCCGCTCATGAGACAATAACCCTGATAAATGCTTCAATAATATTGAAAAAGGAAGAGTATGAGCCATATTCAACGGGAAACGTCTTGCTCTAGGCCGCGATTAAATTCCAACATGGATGCTGATTTATATGGGTATAAATGGGCTCGCGATAATGTCGGGCAATCAGGTGCGACAATCTATCGATTGTATGGGAAGCCCGATGCGCCAGAGTTGTTTCTGAAACATGGCAAAGGTAGCGTTGCCAATGATGTTACAGATGAGATGGTCAGACTAAACTGGCTGACGGAATTTATGCCTCTTCCGACCATCAAGCATTTTATCCGTACTCCTGATGACGCATGGTTACTCACCACTGCGATCCCCGGGAAAACAGCATTCCAGGTATTAGAAGAATATCCTGATTCAGGTGAAAATATTGTTGATGCGCTGGCAGTGTTCCTGCGCCGGTTGCATTCGATTCCTGTTTGTAATTGTCCTTTTAACAGCGACCGCGTATTTCGTCTCGCTCAGGCGCAATCACGAATGAATAACGGTTTGGTTGATGCGAGTGATTTTGATGACGAGCGTAATGGCTGGCCTGTTGAACAAGTCTGGAAAGAAATGCACAAACTTTTGCCATTCTCACCGGATTCAGTCGTCACTCATGGTGATTTCTCACTTGATAACCTTATTTTTGACGAGGGGAAATTAATAGGTTGTATTGATGTTGGACGAGTCGGAATCGCAGACCGATACCAGGATCTTGCCATCCTATGGAACTGCCTCGGTGAGTTTTCTCCTTCATTACAGAAACGGCTTTTTCAAAAATATGGTATTGATAATCCTGATATGAATAAATTGCAGTTTCATTTGATGCTCGATGAGTTTTTCTAACTCGAGGAAGTTCCTATACTTTCTAGAGAATAGGAACTTCGCGGCCGCGCGCAACGCAATTAATGTGAGTTAGCTCACTCATTAGGCACCCCAGGCTTTACACTTTATGCTTCCGGCTCGTATGTTGTGTGGAATTGTGAGCGGATAACAATTTCACACAGGAAACAGCTATGACCATGATTACGGATTCACTGGCCGTCGTTTTACAACGTCGTGACTGGGAAAACCCTGGCGTTACCCAACTTAATCGCCTTGCAGCACATCCCCCTTTCGCCAGCTGGCGTAATAGCGAAGAGGCCCGCACCGATCGCCCTTCCCAACAGTTGCGCAGCCTGAATGGCGAATGGCGCTTTGCCTGGTTTCCGGCACCAGAAGCGGTGCCGGAAAGCTGGCTGGAGTGCGATCTTCCTGAGGCCGATACTGTCGTCGTCCCCTCAAACTGGCAGATGCACGGTTACGATGCGCCCATCTACACCAACGTGACCTATCCCATTACGGTCAATCCGCCGTTTGTTCCCACGGAGAATCCGACGGGTTGTTACTCGCTCACATTTAATGTTGATGAAAGCTGGCTACAGGAAGGCCAGACGCGAATTATTTTTGATGGCGTTAACTCGGCGTTTCATCTGTGGTGCAACGGGCGCTGGGTCGGTTACGGCCAGGACAGTCGTTTGCCGTCTGAATTTGACCTGAGCGCATTTTTACGCGCCGGAGAAAACCGCCTCGCGGTGATGGTGCTGCGCTGGAGTGACGGCAGTTATCTGGAAGATCAGGATATGTGGCGGATGAGCGGCATTTTCCGTGACGTCTCGTTGCTGCATAAACCGACTACACAAATCAGCGATTTCCATGTTGCCACTCGCTTTAATGATGATTTCAGCCGCGCTGTACTGGAGGCTGAAGTTCAGATGTGCGGCGAGTTGCGTGACTACCTACGGGTAACAGTTTCTTTATGGCAGGGTGAAACGCAGGTCGCCAGCGGCACCGCGCCTTTCGGCGGTGAAATTATCGATGAGCGTGGTGGTTATGCCGATCGCGTCACACTACGTCTGAACGTCGAAAACCCGAAACTGTGGAGCGCCGAAATCCCGAATCTCTATCGTGCGGTGGTTGAACTGCACACCGCCGACGGCACGCTGATTGAAGCAGAAGCCTGCGATGTCGGTTTCCGCGAGGTGCGGATTGAAAATGGTCTGCTGCTGCTGAACGGCAAGCCGTTGCTGATTCGAGGCGTTAACCGTCACGAGCATCATCCTCTGCATGGTCAGGTCATGGATGAGCAGACGATGGTGCAGGATATCCTGCTGATGAAGCAGAACAACTTTAACGCCGTGCGCTGTTCGCATTATCCGAACCATCCGCTGTGGTACACGCTGTGCGACCGCTACGGCCTGTATGTGGTGGATGAAGCCAATATTGAAACCCACGGCATGGTGCCAATGAATCGTCTGACCGATGATCCGCGCTGGCTACCGGCGATGAGCGAACGCGTAACGCGAATGGTGCAGCGCGATCGTAATCACCCGAGTGTGATCATCTGGTCGCTGGGGAATGAATCAGGCCACGGCGCTAATCACGACGCGCTGTATCGCTGGATCAAATCTGTCGATCCTTCCCGCCCGGTGCAGTATGAAGGCGGCGGAGCCGACACCACGGCCACCGATATTATTTGCCCGATGTACGCGCGCGTGGATGAAGACCAGCCCTTCCCGGCTGTGCCGAAATGGTCCATCAAAAAATGGCTTTCGCTACCTGGAGAGACGCGCCCGCTGATCCTTTGCGAATACGCCCACGCGATGGGTAACAGTCTTGGCGGTTTCGCTAAATACTGGCAGGCGTTTCGTCAGTATCCCCGTTTACAGGGCGGCTTCGTCTGGGACTGGGTGGATCAGTCGCTGATTAAATATGATGAAAACGGCAACCCGTGGTCGGCTTACGGCGGTGATTTTGGCGATACGCCGAACGATCGCCAGTTCTGTATGAACGGTCTGGTCTTTGCCGACCGCACGCCGCATCCAGCGCTGACGGAAGCAAAACACCAGCAGCAGTTTTTCCAGTTCCGTTTATCCGGGCAAACCATCGAAGTGACCAGCGAATACCTGTTCCGTCATAGCGATAACGAGCTCCTGCACTGGATGGTGGCGCTGGATGGTAAGCCGCTGGCAAGCGGTGAAGTGCCTCTGGATGTCGCTCCACAAGGTAAACAGTTGATTGAACTGCCTGAACTACCGCAGCCGGAGAGCGCCGGGCAACTCTGGCTCACAGTACGCGTAGTGCAACCGAACGCGACCGCATGGTCAGAAGCCGGGCACATCAGCGCCTGGCAGCAGTGGCGTCTGGCGGAAAACCTCAGTGTGACGCTCCCCGCCGCGTCCCACGCCATCCCGCATCTGACCACCAGCGAAATGGATTTTTGCATCGAGCTGGGTAATAAGCGTTGGCAATTTAACCGCCAGTCAGGCTTTCTTTCACAGATGTGGATTGGCGATAAAAAACAACTGCTGACGCCGCTGCGCGATCAGTTCACCCGTGCACCGCTGGATAACGACATTGGCGTAAGTGAAGCGACCCGCATTGACCCTAACGCCTGGGTCGAACGCTGGAAGGCGGCGGGCCATTACCAGGCCGAAGCAGCGTTGTTGCAGTGCACGGCAGATACACTTGCTGATGCGGTGCTGATTACGACCGCTCACGCGTGGCAGCATCAGGGGAAAACCTTATTTATCAGCCGGAAAACCTACCGGATTGATGGTAGTGGTCAAATGGCGATTACCGTTGATGTTGAAGTGGCGAGCGATACACCGCATCCGGCGCGGATTGGCCTGAACTGCCAGCTGGCGCAGGTAGCAGAGCGGGTAAACTGGCTCGGATTAGGGCCGCAAGAAAACTATCCCGACCGCCTTACTGCCGCCTGTTTTGACCGCTGGGATCTGCCATTGTCAGACATGTATACCCCGTACGTCTTCCCGAGCGAAAACGGTCTGCGCTGCGGGACGCGCGAATTGAATTATGGCCCACACCAGTGGCGCGGCGACTTCCAGTTCAACATCAGCCGCTACAGTCAACAGCAACTGATGGAAACCAGCCATCGCCATCTGCTGCACGCGGAAGAAGGCACATGGCTGAATATCGACGGTTTCCATATGGGGATTGGTGGCGACGACTCCTGGAGCCCGTCAGTATCGGCGGAATTCCAGCTGAGCGCCGGTCGCTACCATTACCAGTTGGTCTGGTGTCAAAAATAAGCGGCCGCTCAAGACGCCTTCGCCAACTCCTTCACCAGAGGTAGCATTATCCGCATAACGTCACGGCAGCGACGTTCTATTCTTCCAGGAAGAGCCTTATCAATATGTTGGTGATTATCCAGTCTTACGTCATGCCAGCTATTTCCCGCCGGGAAGGCAGGTGTTTTTGCGCGTTGCTGATAACCATCCTTATTCCCAAGATTCCAGTTAGTCGCTTCCACCGAAAGTACAGCAATGCCCGCTTTGTCGAATATTTCTGCGTCATTACAACACCCAGTGCCTTTCGGATAATTTTTATTCAAACCCGGATTGGTCGTTGCGGCTATTCCGTGACTGCGCGCAATTGCCAGCGCCCTGTCGCGCGTTAATTTCCTTACTGCCTCAGGGGTTTTTACACCGCTGTTGAAAAAGCTT

**Synthetic CRISPR cassette of Cr2.1::IE1**

CTGCAGCGTGCTTGCCGCTGGAGAAATACAACCGCCGGCCCCACCTGAAGATGCACAGCCTGTTGCCATTCCGCTTCCTGTTTCACTGGGAGATGCAGGCCATCGGAGTAGCTGAAATGAGTATGTTGGTCGTGGTCACTGAAAATGTACCTCCGCGCTTACGAGGCAGATTAGCCATCTGGTTGTTGGAGGTACGTGCAGGGGTATATGTAGGTGATGTATCCGCAAAAATTCGTGAAATGATCTGGGAACAAATAGCTGGACTGGCGGAAGAAGGCAATGTAGTGATGGCATGGGCAACGAATACGGAAACGGGATTTGAGTTCCAGACATTTGGGTTAAACAGGCGTACCCCGGTAGATTTGGATGGTTTAAGGTTGGTGTCTTTTTTACCTGTTTGACCATGGAAACAAAGAATTAGCTGATCTTTAATAATAAGGAAATGTTACATTAAGGTTGGTGGGTTGTTTTTATGGGAAAAAATGCTTTAAGAACAAATGTATACTTTTAGAGAGTTCCCCGCGCCAGCGGGGATAAACCGTGGGATGCCTACCGCAAGCAGCTTGGCCTGAAGAGTTCCCCGCGCCAGCGGGGATAAACCGGACCGACGTCCACCGAATTCGCCGAACACGCTGAGTTCCCCGCGCCAGCGGGGATAAACCGCCGCTTTCGCGGCATATTCTCGCCGTCAAAAAGAGTTCCCCGCGCCAGCGGGGATAAACCGTTTGGATCGGGTCTGGATCCCTAGGTGCCTGGGAGTTCCCCGCGCCAGCGGGGATAAACCGGCCCATTCCAAAGGGTCAGGTTCGGTGGTTGTGAGTTCCCCGCGCCAGCGGGGATAAACCGGACTTACATTTATAATGCATAATAGGAATCACGAGTTCCCCGCGCCAGCGGGGATAAACCGACGGCTTCATGTTCCTTGTGATGGGCTTTAGGGAGTTCCCCGCGCCAGCGGGGATAAACCGCAGCTCCCATTTTCAAACCCAGGTACCGAAGTTCCTATACTTTCTAGAGAATAGGAACTTCGCATGCCTATTTGTTTATTTTTCTAAATACATTCAAATATGTATCCGCTCATGAGACAATAACCCTGATAAATGCTTCAATAATATTGAAAAAGGAAGAGTATGAGCCATATTCAACGGGAAACGTCTTGCTCTAGGCCGCGATTAAATTCCAACATGGATGCTGATTTATATGGGTATAAATGGGCTCGCGATAATGTCGGGCAATCAGGTGCGACAATCTATCGATTGTATGGGAAGCCCGATGCGCCAGAGTTGTTTCTGAAACATGGCAAAGGTAGCGTTGCCAATGATGTTACAGATGAGATGGTCAGACTAAACTGGCTGACGGAATTTATGCCTCTTCCGACCATCAAGCATTTTATCCGTACTCCTGATGACGCATGGTTACTCACCACTGCGATCCCCGGGAAAACAGCATTCCAGGTATTAGAAGAATATCCTGATTCAGGTGAAAATATTGTTGATGCGCTGGCAGTGTTCCTGCGCCGGTTGCATTCGATTCCTGTTTGTAATTGTCCTTTTAACAGCGACCGCGTATTTCGTCTCGCTCAGGCGCAATCACGAATGAATAACGGTTTGGTTGATGCGAGTGATTTTGATGACGAGCGTAATGGCTGGCCTGTTGAACAAGTCTGGAAAGAAATGCACAAACTTTTGCCATTCTCACCGGATTCAGTCGTCACTCATGGTGATTTCTCACTTGATAACCTTATTTTTGACGAGGGGAAATTAATAGGTTGTATTGATGTTGGACGAGTCGGAATCGCAGACCGATACCAGGATCTTGCCATCCTATGGAACTGCCTCGGTGAGTTTTCTCCTTCATTACAGAAACGGCTTTTTCAAAAATATGGTATTGATAATCCTGATATGAATAAATTGCAGTTTCATTTGATGCTCGATGAGTTTTTCTAACTCGAGGAAGTTCCTATACTTTCTAGAGAATAGGAACTTCGCGGCCGCTCAAGACGCCTTCGCCAACTCCTTCACCAGAGGTAGCATTATCCGCATAACGTCACGGCAGCGACGTTCTATTCTTCCAGGAAGAGCCTTATCAATATGTTGGTGATTATCCAGTCTTACGTCATGCCAGCTATTTCCCGCCGGGAAGGCAGGTGTTTTTGCGCGTTGCTGATAACCATCCTTATTCCCAAGATTCCAGTTAGTCGCTTCCACCGAAAGTACAGCAATGCCCGCTTTGTCGAATATTTCTGCGTCATTACAACACCCAGTGCCTTTCGGATAATTTTTATTCAAACCCGGATTGGTCGTTGCGGCTATTCCGTGACTGCGCGCAATTGCCAGCGCCCTGTCGCGCGTTAATTTCCTTACTGCCTCAGGGGTTTTTACACCGCTGTTGAAAAAGCTT

**Synthetic CRISPR cassette of Cr2.1::IE1 *ccdA***CTGCAGCGTGCTTGCCGCTGGAGAAATACAACCGCCGGCCCCACCTGAAGATGCACAGCCTGTTGCCATTCCGCTTCCTGTTTCACTGGGAGATGCAGGCCATCGGAGTAGCTGAAATGAGTATGTTGGTCGTGGTCACTGAAAATGTACCTCCGCGCTTACGAGGCAGATTAGCCATCTGGTTGTTGGAGGTACGTGCAGGGGTATATGTAGGTGATGTATCCGCAAAAATTCGTGAAATGATCTGGGAACAAATAGCTGGACTGGCGGAAGAAGGCAATGTAGTGATGGCATGGGCAACGAATACGGAAACGGGATTTGAGTTCCAGACATTTGGGTTAAACAGGCGTACCCCGGTAGATTTGGATGGTTTAAGGTTGGTGTCTTTTTTACCTGTTTGACCATGGAAACAAAGAATTAGCTGATCTTTAATAATAAGGAAATGTTACATTAAGGTTGGTGGGTTGTTTTTATGGGAAAAAATGCTTTAAGAACAAATGTATACTTTTAGAGAGTTCCCCGCGCCAGCGGGGATAAACCGTGGGATGCCTACCGCAAGCAGCTTGGCCTGAAGAGTTCCCCGCGCCAGCGGGGATAAACCGGACCGACGTCCACCGAATTCGCCGAACACGCTGAGTTCCCCGCGCCAGCGGGGATAAACCGCCGCTTTCGCGGCATATTCTCGCCGTCAAAAAGAGTTCCCCGCGCCAGCGGGGATAAACCGTTTGGATCGGGTCTGGATCCCTAGGTGCCTGGGAGTTCCCCGCGCCAGCGGGGATAAACCGGCCCATTCCAAAGGGTCAGGTTCGGTGGTTGTGAGTTCCCCGCGCCAGCGGGGATAAACCGGACTTACATTTATAATGCATAATAGGAATCACGAGTTCCCCGCGCCAGCGGGGATAAACCGACGGCTTCATGTTCCTTGTGATGGGCTTTAGGGAGTTCCCCGCGCCAGCGGGGATAAACCGCAGCTCCCATTTTCAAACCCAGGTACCCCATACGAAACGGGAATGCGGTAATTACGCTTTGTTTTTATAAGTCAGATTTTAATTTTTATTGGTTAACATAACGAAAGGTAAAATACATAAGGCTTACTAAAAGCCAGATAACAGTATGCGTATTTGCGCGCTGATTTTTGCGGTATAAGAATATATACTGATATGTATACCCGAAGTATGTCAAAAAGAGGTGTGCTATGAAGCAGCGTATTACAGTGACAGTTGACAGCGACAGCTATCAGTTGCTCAAGGCATATGATGTCAATATCTCCGGTCTGGTAAGCACAACCATGCAGAATGAAGCCCGTCGTCTGCGTGCCGAACGCTGGAAAGCGGAAAATCAGGAAGGGATGGCTGAGGTCGCCCGGTTTATTGAAATGAACGGCTCTTTTGCTGACGAGAACAGGGACTGGTGAGGTACCGAAGTTCCTATACTTTCTAGAGAATAGGAACTTCGCATGCCTATTTGTTTATTTTTCTAAATACATTCAAATATGTATCCGCTCATGAGACAATAACCCTGATAAATGCTTCAATAATATTGAAAAAGGAAGAGTATGAGCCATATTCAACGGGAAACGTCTTGCTCTAGGCCGCGATTAAATTCCAACATGGATGCTGATTTATATGGGTATAAATGGGCTCGCGATAATGTCGGGCAATCAGGTGCGACAATCTATCGATTGTATGGGAAGCCCGATGCGCCAGAGTTGTTTCTGAAACATGGCAAAGGTAGCGTTGCCAATGATGTTACAGATGAGATGGTCAGACTAAACTGGCTGACGGAATTTATGCCTCTTCCGACCATCAAGCATTTTATCCGTACTCCTGATGACGCATGGTTACTCACCACTGCGATCCCCGGGAAAACAGCATTCCAGGTATTAGAAGAATATCCTGATTCAGGTGAAAATATTGTTGATGCGCTGGCAGTGTTCCTGCGCCGGTTGCATTCGATTCCTGTTTGTAATTGTCCTTTTAACAGCGACCGCGTATTTCGTCTCGCTCAGGCGCAATCACGAATGAATAACGGTTTGGTTGATGCGAGTGATTTTGATGACGAGCGTAATGGCTGGCCTGTTGAACAAGTCTGGAAAGAAATGCACAAACTTTTGCCATTCTCACCGGATTCAGTCGTCACTCATGGTGATTTCTCACTTGATAACCTTATTTTTGACGAGGGGAAATTAATAGGTTGTATTGATGTTGGACGAGTCGGAATCGCAGACCGATACCAGGATCTTGCCATCCTATGGAACTGCCTCGGTGAGTTTTCTCCTTCATTACAGAAACGGCTTTTTCAAAAATATGGTATTGATAATCCTGATATGAATAAATTGCAGTTTCATTTGATGCTCGATGAGTTTTTCTAACTCGAGGAAGTTCCTATACTTTCTAGAGAATAGGAACTTCGCGGCCGCTCAAGACGCCTTCGCCAACTCCTTCACCAGAGGTAGCATTATCCGCATAACGTCACGGCAGCGACGTTCTATTCTTCCAGGAAGAGCCTTATCAATATGTTGGTGATTATCCAGTCTTACGTCATGCCAGCTATTTCCCGCCGGGAAGGCAGGTGTTTTTGCGCGTTGCTGATAACCATCCTTATTCCCAAGATTCCAGTTAGTCGCTTCCACCGAAAGTACAGCAATGCCCGCTTTGTCGAATATTTCTGCGTCATTACAACACCCAGTGCCTTTCGGATAATTTTTATTCAAACCCGGATTGGTCGTTGCGGCTATTCCGTGACTGCGCGCAATTGCCAGCGCCCTGTCGCGCGTTAATTTCCTTACTGCCTCAGGGGTTTTTACACCGCTGTTGAAAAAGCTT
